# Impact of long-lasting, insecticidal nets on anaemia and prevalence of *Plasmodium falciparum* among children under five years in areas with highly resistant malaria vectors

**DOI:** 10.1186/1475-2875-13-76

**Published:** 2014-03-01

**Authors:** Filémon T Tokponnon, Aurore Hounto Ogouyémi, Yolande Sissinto, Arthur Sovi, Virgile Gnanguenon, Sylvie Cornélie, Adicath Adéola Adéothy, Razaki Ossè, Abel Wakpo, Dina Gbénou, Mariam Oke, Dorothée Kinde-Gazard, Immo Kleinschmidt, Martin C Akogbeto, Achille Massougbodji

**Affiliations:** 1 National Malaria Control Programme, Cotonou, Benin; 2 Ministry of Health, Cotonou, Benin; 3 Faculté des Sciences de la Santé de l’Université d’Abomey Calavi, Calavi, Benin; 4 World Health Organization, Cotonou, Benin; 5 Faculte des Sciences et Techniques de l’Université d’Abomey-Calavi, Calavi, Benin; 6 Centre de Recherche Entomologique de Cotonou (CREC), Cotonou, Benin; 7 Institut de Recherche pour le Développement, MIVEGEC, UM1-CNRS 5290-IRD 224, Cotonou, Benin; 8 Department of Infectious Disease Epidemiology, London School of Hygiene and Tropical Medicine, London, UK

**Keywords:** Malaria, Prevalence of *Plasmodium falciparum*, Anaemia, Resistance, LLINs

## Abstract

**Background:**

The widespread use of insecticide-treated nets (LLINs) leads to the development of vector resistance to insecticide. This resistance can reduce the effectiveness of LLIN-based interventions and perhaps reverse progress in reducing malaria morbidity. To prevent such difficulty, it is important to know the real impact of resistance in the effectiveness of mosquito nets. Therefore, an assessment of LLIN efficacy was conducted in malaria prevention among children in high and low resistance areas.

**Methods:**

The study was conducted in four rural districts and included 32 villages categorized as low or high resistance areas in Plateau Department, south-western Benin. Larvae collection was conducted to measure vector susceptibility to deltamethrin and knockdown resistance (*kdr*) frequency. In each resistance area, around 500 children were selected to measure the prevalence of malaria infection as well as the prevalence of anaemia associated with the use of LLINs.

**Results:**

Observed mortalities of *Anopheles gambiae s.s* population exposed to deltamethrin ranged from 19 to 96%. Knockdown resistance frequency was between 38 and 84%. The prevalence of malaria infection in children under five years was 22.4% (19.9-25.1). This prevalence was 17.3% (14.2-20.9) in areas of high resistance and 27.1% (23.5-31.1) in areas of low resistance (p = 0.04). Eight on ten children that were aged six - 30 months against seven on ten of those aged 31–59 months were anaemic. The anaemia observed in the six to 30-month old children was significantly higher than in the 31–59 month old children (p = 0.00) but no difference associated with resistance areas was observed (p = 0.35). The net use rate was 71%. The risk of having malaria was significantly reduced (p < 0.05) with LLIN use in both low and high resistance areas. The preventive effect of LLINs in high resistance areas was 60% (95% CI: 40–70), and was significantly higher than that observed in low resistance areas (p < 0.05).

**Conclusion:**

The results of this study showed that the resistance of malaria vectors seems to date not have affected the impact of LLINs and the use of LLINs was highly associated with reduced malaria prevalence irrespective of resistance.

## Background

Malaria remains a deadly endemic disease and a growing concern around the world [1]. Its control is based on both preventing transmission and promptly treating infection. Insecticide-treated nets (LLINs) are effective tools for malaria prevention and can significantly reduce severe disease and mortality due to malaria, especially among children aged under five years in endemic areas [2].

LLINs have a community effect by reducing the longevity of malaria vectors [3]. Many countries in the past decade have made significant progress in preventing malaria by largely focusing on vector control through LLINs and indoor residual spraying (IRS) of insecticides. Several strategies, including free distribution to target groups [4,5] and free, universal, population-based distribution campaigns, target an entire population at risk [4,6]. It is estimated that between 2000 and 2010, LLINs has saved more than 908,000 lives, and since 2006, prevented three-quarters of deaths due to malaria [7]. However, the widespread use of LLINs leads to the development of vector resistance to insecticide. This insecticide resistance can reduce the effectiveness of these interventions and perhaps reverse progress in reducing malaria morbidity [8]. Although resistance may be inevitable with effective control programmes, new strategies must be developed to reduce the development and spread of insecticide resistance and preserve the effectiveness of currently available insecticides and malaria control interventions. It is obvious that increasing the level of resistance corresponds to a decrease in the effectiveness of vector control strategies implementation [9].

Benin is currently involved in a national campaign of free distribution of LLINs for universal access. In July 2011, an average of 86% of households were covered throughout the country [10]. The first cases of resistant vectors were noted before 2000 in several localities [11-14]. With the massive use of insecticides in both public health and agriculture [15] the level of resistance has considerably increased and in localities where vectors were susceptible to becoming resistant [12].

Recent studies show that pyrethroid treatments failed to kill resistant vectors in experimental trials of LLINs where the main brands of nets were used (Permanet 2.0 and Olyset net) [16]. Household protection with holed LLINs was lost in areas where vectors were resistant to pyrethroids [17] and an average of five *Anopheles gambiae sensu lato (s.l*.) by night can enter torn nets at a proportionate hole index of 276 [18]. Additionally, studies showed that reductions in haemoglobin levels in endemic areas were created by malaria infections [19,20], thus, it was not possible to clearly separate the effects of parasites from those of anaemia on the resulting measurements of vectors in the transmission. Resistance is on the rise and that is a real threat to the vector control interventions that are currently used and that in high coverage have show to lead to excellent results. But there is very little data at the moment that helps us judge if this resistance translates to reduced malaria indicators.

Therefore, it was important to assess the impact of vector resistance and LLIN use on malaria prevalence in the community. The objectives of this study were to: i) determine *An. gambiae* susceptibility to deltamethrin and knockdown resistance (*Kdr*) frequency; ii) assess the prevalence of malaria infection; iii) measure the LLIN use rate; iv) assess the prevalence of anaemia among children aged six to 59 months, and v) compare the different indicators in low and high insecticides resistance areas.

## Methods

### Study area

The study was conducted in four rural districts belonging to two health regions (Ifangni-Sakete and Pobe-Ketou) in Plateau Department, south-western Benin. This area is characterized by two rainy seasons (April to July and September to November) and two dry seasons (December to March and August to September). The selection of this Department was based on its geographic accessibility and the high use of mosquito nets by children aged under five years. Entomological surveys conducted in Plateau Department showed that there are two categories of localities: those with low resistance and those with high resistance by vectors to pyrethroids [13]. According to the report of the LLIN distribution campaign, 85.5% of households received an LLIN, with an average of 2.70 LLINs/household [10]. The four districts selected were Ifangni, Sakete, Ketou, and Pobe.

**Figure 1:**
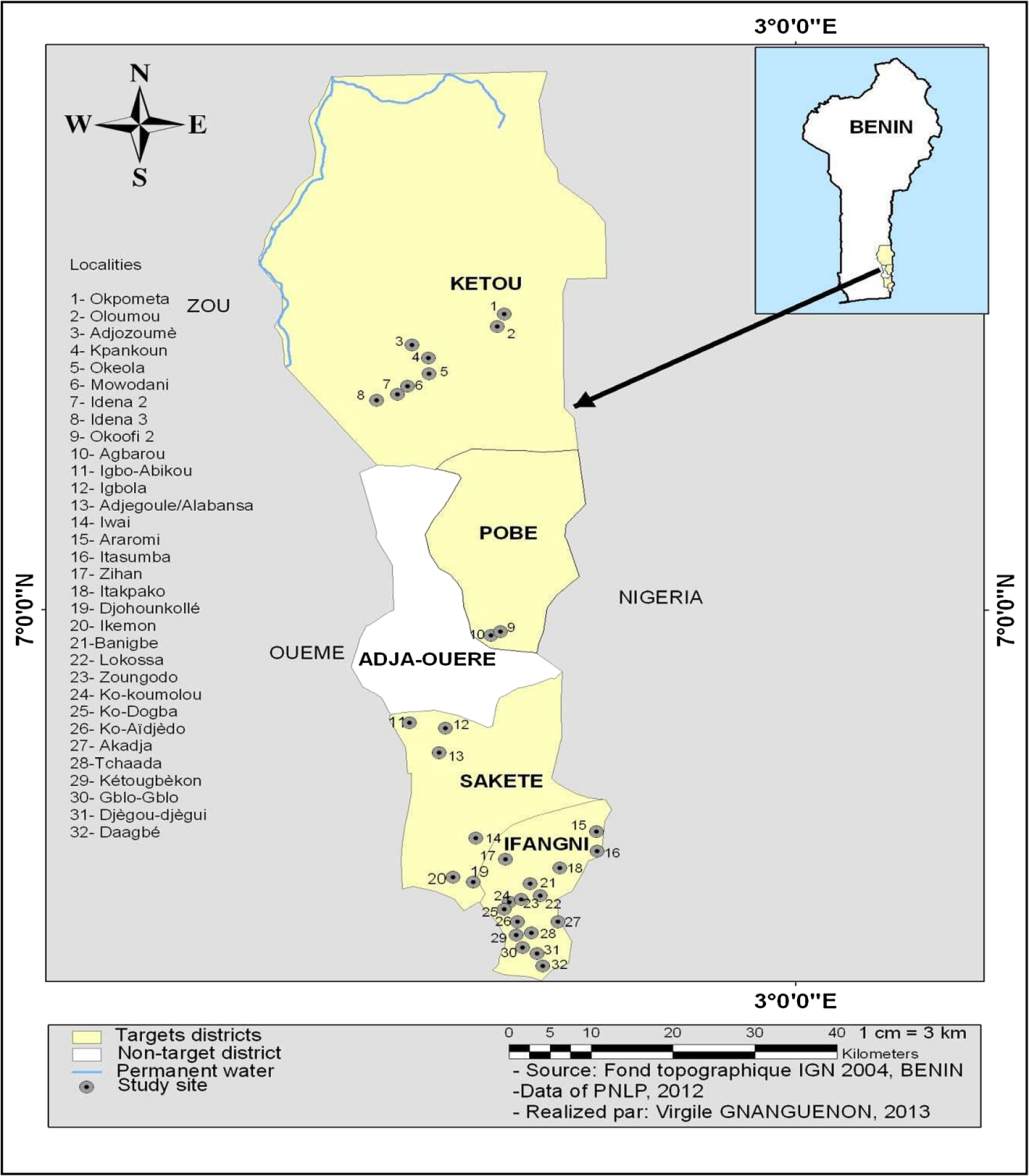
Study sites.

Ifangni district is located at 2°43'14"E and 6°38'56"N; its area is 242 sq km representing 7.28% of Plateau territory. Sakete is located at 2°39'7"E and 6°46'3"N, covering an area of 432 sq km, and represents 13.29% of Plateau territory. Ketou is at 2°36'4"E and 7°27'21"N, with an area of 1,775 sq km, representing 54.38% of Plateau territory. Pobe is at 2°41'51"E and 7°5'12"N and has an area of 400 sq km that represents 11% of Plateau territory. Thirty-two rural villages were selected through the four districts (Figure 1).

### Study design

Before the study began, WHO susceptibility tests were performed on *An. gambiae* using deltamethrin to select the villages (clusters) where activities were held. Table 1 shows the distribution of the villages based on the mortalities observed with deltamethrin in 2011. Due to the absence of an area where *An. gambiae* are fully susceptible to pyrethroids in Benin (Djègbè, pers comm), criteria were used to categorize the level of resistance. “R+++ area” was called an area where the observed mortality was between 0 and 60% and “R + area” an area where the observed mortality ranged 80 to 100%. These two areas were identified based on baseline resistance data collected in Plateau Department (Djègbè, pers comm). Thus, 16 villages of high resistance and 16 villages of low resistance were selected to host the work. Note that most of the villages included in the study were located at Ifangni district, and mainly low resistance villages (Table 1).

**Table 1:** Distribution of the clusters based on the low or high resistance status in 2011 according the districts

**Districts**	**IFANGNI (16)**	**SAKETE (06)**	**POBE (02)**	**KETOU (08)**
	Itassoumba*	Iwai*	Okoofi2^#^	Adjozoumè*
	Itakpako*	Igbola^#^	Agbarou*	Mowodani^#^
	Ko Koumolou*	Ikémon^#^		Idéna2^#^
	Ko Aidjèdo*	Igbo-abikou^#^		Idena3^#^
	Kétougbèkon*	Alabansa^#^		Kpankoun^#^
	Lokossa*	Djohounkollé*		Okpometa^#^
**Clusters**	Ko-Dogba*			Okeola^#^
	Zoungodo*			Omou*
	Zihan*			
	Araromi*			
	Daagbé*			
	Akadja^#^			
	Tchaada^#^			
	Banigbé centre^#^			
	Djégou-Djègi^#^			
	GbloGblo^#^			
	Zoungodo*			

*Low resistance area (R^+^) (80-100%); ^#^High resistance area (R^+++^) (0-60%).

Each cluster (village) was composed of several hamlets and included a minimum of 100 children under five years old. Cross-sectional surveys were conducted in each cluster in May to August 2012, during the high malaria transmission period. The surveys covered the targeted groups in different villages. In each cluster, 40 children less than five years old, 25 pregnant women, 30 children over five years old, and adult heads of household were selected. The results reported here were for those of children under five years old and were analysed with vector resistance data from for the same period.

Larvae collection of *An. gambiae s.l.* in seven villages was not productive during the study period to observe resistance level of the mosquitoes. These villages were dropped from data analysis. For this, the work was continued in 25 villages. Before starting the survey, training of investigators (laboratory technicians, nurses and other staff) followed by a pretest questionnaire was performed. In the field, after the approval of the chiefs of villages, investigators sampled randomly the survey households by selecting one house in every two in each village. Interviews were conducted through a questionnaire provided by investigators, and was followed by the realization of blood smear and haemoglobin test by laboratory technicians. Information on the use of LLINs by households was verified during the investigation. Indeed, in each village, about a questionnaire, households were interviewed about LLINs ownership and their use. People who use them are those who reported having slept under LLINs the previous night of the survey.

### Data collection

#### *Collection of Anopheles gambiae larvae*

Larvae of *An. gambiae* were collected in the all villages by the “dipping” method, which involves capturing mosquito larvae directly in their productive breeding sites using a simple ladle. These breeding sites were the puddles and located near the differents villages. The larvae and pupae were kept separately in labelled bottles and were reared in the insectarium of Centre de Recherche Entomologique de Cotonou (CREC) until they emerged into adults mosquitoes. Females aged from two to five days were used for WHO susceptibility bioassay under laboratory conditions (25°C ± 2°C and 80 ± 4% relative humidity).

### Susceptibility of *Anopheles gambiae* to deltamethrin

Phenotypic determination of the level of resistance was done using susceptibility tests (bioassays cylinder tube) according to WHO guidelines [21]. This susceptibility test was performed using unfed females of *An. gambiae s.l,* aged two to five days. The bioassays were carried out with impregnated papers of deltamethrin (0.05%). Four batches of twenty-five females were introduced into treatment tubes for 60 min. Two batches exposed to untreated papers were used as control. The number of knocked-down mosquitoes was recorded every 10 min during the period of exposure. After 60 min exposure, the mosquitoes were transferred into observation tubes and were fed with 10% honey solution then maintained in observation for 24 hours. At the end of the observation period, mortality rate was calculated. According to WHO technical guidelines [21], a mortality rate higher than 97% means that the population of mosquitoes tested is susceptible; a mortality rate between 90 and 97% means there is a suspicion of resistance and a mortality rate lower than 90% means the mosquito population tested is resistant. After the tests, the dead and living mosquitoes were conserved separately on silica gel and stored at -20°C for molecular characterization by PCR.

### Characterization of the populations of *Anopheles gambiae* by PCR: species, molecular form and *Kdr* Leu-phe mutation

Approximately 16–126 females of *An. gambiae* from each village resulting from the susceptibility tests were analysed by PCR. DNA from control (non-exposed) mosquitoes was extracted individually by CTAB technique. Species among *An. gambiae* complex and molecular form were determined by PCR [22,23]. *Kdr* mutation was determined by HOLA technique described by Lynd *et al.*[24]. This technique allowed the detecting of *Kdr* mutation.

### Realization of blood smear and thick film

Thick film and blood smear were performed in villages by laboratory technicians from blood collected by phlebotomy after puncture children’s finger by lancets. The slides were identified and sprawl were dried and stored in boxes slides for their delivery to the laboratory.

### Laboratory examination of slides

The slides were brought to the laboratory for a double reading by trained technicians. Parasitological infection was detected on 10% Giemsa-stained thick smears. A sexual stage of each *Plasmodium* species was counted in the blood volume occupied by 200 leucocytes and parasite density was calculated by assuming 8,000 leucocytes/μL of blood. Thick smears from each village were read by the same experienced technician, under the supervision of a parasitologist. The readings of the two technicians were also compared on the same set of blood samples. Their estimations of parasite detection and parasite density did not differ significantly. Crosscheck quality control was done on a randomly selected sample representing 10% of all thick smears.

### Determination of haemoglobin

The haemoglobin concentration (g/dL) was done by Hemo-Control EKF Diagnostic analiser that used undiluted blood. Potassium cyanide used in the reference method is replaced by sodium azide. The haemo-drive control uses pits with a short light path containing three reagents: sodium deoxycholate, sodium nitrate and sodium azide. Only 10 μL of capillary blood are needed. When the microbasin is filled by capillary action, it must be adapted to fit into the haemo-control part and fold the tab. The rate of haemoglobin is obtained within 25–60 seconds.

### Statistical analyses

Demographic, biological and entomological data were double-entered independently in the Epi database. Parasitological and clinical data were analyzed using the survey command (SPSS16.0). Parasitological data were analysed separately in terms of prevalence of *Plasmodium falciparum* asexual blood forms, density of *P. falciparum* asexual blood forms in parasite-positive blood thick films. The prevalence of asymptomatic malaria infections was analysed as a binomial response by using a logistic regression model.

To measure the strength of the association between the explanatory factors (use of mosquito nets by children the day before the survey, status of low or high vector resistance in the villages), the prevalence of infection and the prevalence of anemia, the ratio of the coast or odds ratio (OR) was calculated. Allelic frequencies of *Kdr* mutation were compared with GENEPOP software. Differences were considered significant for p < 0.05.

### Ethical clearance

This study was planned and approved by the Ministry of Health, Benin. The protocol was also reviewed and approved by National Ethics Committee for Health Research at the Ministry of Health, Benin. A briefing note indicating the objectives of the study, the advantages and disadvantages was given to the respondents in order to obtain consent. Confidentiality was respected and questionnaires were anonymous.

## Results

### Mortality rates, molecular form and knockdown resistance of *Anopheles gambiae*

The observed mortality of vector population exposed to deltamethrin ranged from 19-96% (Table 2). The results do not allow a fair distribution of R + and R+++ areas according to the criteria of departure. Only three of the twenty-five villages positive in the collection of larvae obey the criterion of R+++. Thus, in order to standardize the analysis and have two groups of localities based on the level of resistance as discriminatory variable, the median of mortality rate was determined. The median mortality rate was 79% (74.0-83.9) CI 95%. Therefore, 12 localities of high resistance and 13 localities of low resistance were distinguished.

**Table 2:** **Results cluster specific phenotype data 2012 and *Kdr *** **frequencies**

**Cluster name**	**Country specific resistance classification**	**No exposed**	**No killed 24 hours after 1-hr exposure**	**Mortality (%)**	**Samples size (2n)**	**Frequency (%) *Kdr***
**Banigbé**	**R+++**	123	24	19.51	102	74.5
**Kokoumolou**	**R+++**	172	96	55.81	80	63.8
**Agbarou**	**R+++**	211	126	59.72	72	65.3
**Araromi**	**R+++**	141	89	63.12	98	61.2
**Ko-Dogba**	**R+++**	277	181	65.34	124	68.5
**Mowodani**	**R+++**	118	86	72.88	24	66.7
**Igbo-Abikou**	**R+++**	194	143	73.71	60	76.7
**Idena3**	**R+++**	199	147	73.87	126	68.3
**Alabansa**	**R+++**	192	142	73.96	100	72,0
**Tchaada**	**R+++**	225	167	74.22	98	83.7
**Adjozoume**	**R+++**	251	188	74.90	74	66.2
**Iwaï**	**R+++**	45	35	77.78	46	63.0
**Total (R+++)**		**2,148**	**1,424**	**65.40**		**69.1**
**Djohounkolé**	**R+**	257	203	79.00	112	69.6
**Kétougbékon**	**R+**	349	278	79.66	58	62.1
**Lokossa**	**R+**	327	261	79.82	114	65.8
**Itakpako**	**R+**	297	238	80.13	42	54.8
**Igbola**	**R+**	196	159	81.12	54	77.8
**Ita-soumba**	**R+**	352	290	82.39	48	66.7
**Ko-Aïdjedo**	**R+**	302	250	82.78	46	52,2
**Zihan**	**R+**	342	287	83.92	70	38.6
**Gblo-Gblo**	**R+**	50	42	84.00	40	62.5
**Okéola**	**R+**	481	430	89.40	104	74.0
**Idena2**	**R+**	651	618	94,93	138	69.6
**Kpankoun**	**R+**	50	48	96.00	16	50.0
**Daagbe**	**R+**	26	25	96.15	28	60.7
**Total (R+)**		**3,680**	**3129**	**85.33**		**61.8**

**R *+++ *:** high resistance **R *+ *:** low resistance.

**Figure 2:**
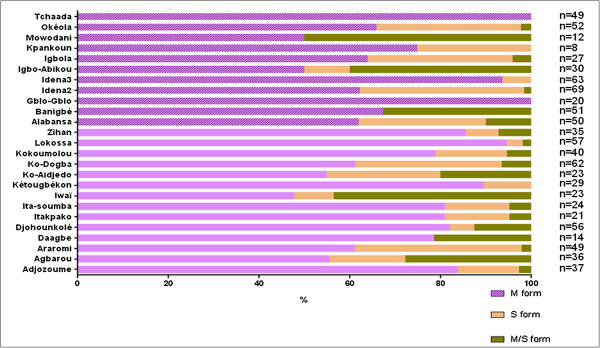
**Molecular form of *Anopheles gambiae *** **collected by cluster.**

Knock down resistance frequencies was between 38-84% (Table 2). All *An. gambiae s.l.* collected was *An. gambiae sensu stricto (s.s)* (100%). The results of molecular form identification showed that M and S were present in most of the villages (Figure 2). *Anopheles gambiae s.s.* collected from Gblo-gblo and Tchaada villages were only M form. Some hybrids M/S of *An. gambiae s.s.* were also found in several villages (Figure 2). The *kdr* mutation was found in both M and S molecular form of *An. gambiae s.s.,* but their frequencies varied according to villages. The S form was found in very small proportion (Figure 2).

### Population description, net use, prevalence of infection, and anaemia

A total of 1,000 children aged six to 59 months from the 25 clusters were tested for *P. falciparum* malaria infection. In each cluster (with low or high resistance), around 40 children were selected. The average age of the children included in the study was 27 months. In the low resistance localities the average age was 26 months against 28 months in high resistance areas (Table 3). Of the households selected in the 25 clusters, 89% had at least one LLIN. 71% of children followed slept under LLIN the night before the survey. In the low resistance area, the proportion of children sleeping under LLIN was 74% against 68% in the high resistance area (Table 3).

**Table 3:** Characteristics of the children used in the analysis and all children tested in the survey

	**Low resistance area (13 clusters)**	**High resistance area (12 clusters)**
**Number of children**	520	480
**Prevalence of malaria (%)**	27.1 (23.5-31.1)	17.3 (14.2-20.9)
**Average age (months)**	26.5 (25.3-27.8)	28.4 (27.3-29.6)
**Slept under net (%)**	74.1 (70.1-77.6)	68.0 (63.6-71.9)
**Mean of haemoglobin rate**	9.5 (9.4-9.7)	9.2 (9.1-9.3)

**Figure 3:**
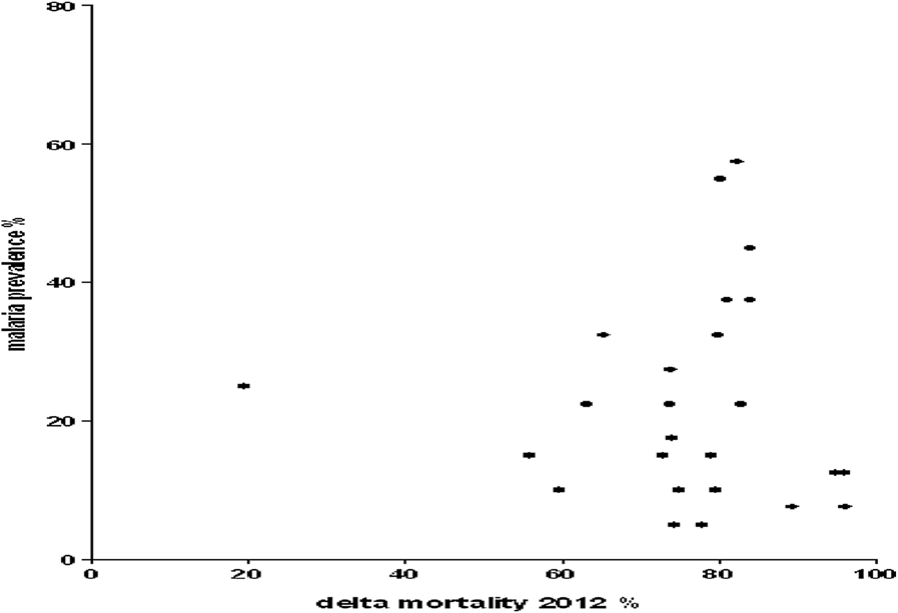
Malaria prevalence for each cluster by deltamethrin mortality.

The prevalence of malaria infection in children aged under five years in the community was 22.4% (19.9-25.1) (Table 4). This prevalence was 17.3% (14.2-20.9) in areas of high resistance and 27.1% (23.5-31.1) in areas of low resistance (p = 0.04). There was more infection to *P. falciparum* in areas that showed higher mortality to deltamethrin. However, the villages taken separately showed no link between the prevalence of *P. falciparum* infection and mortality deltamethrin (Figure 3).

**Table 4:** **Prevalence of *Plasmodium falciparum *** **in low and high resistance areas**

**Cluster name**	**Country specific resistance classification**	**Number of blood smears**	**Number of positive blood smears**	**Prevalence of *P. f *** **(%)**
**Banigbe**	**R+++**	40	10	25.0
**Kokoumolou**	**R+++**	40	6	15.0
**Agbarou**	**R+++**	40	4	10.0
**Araromi**	**R+++**	40	9	22.5
**Ko-Dogba**	**R+++**	40	13	32.5
**Mowodani**	**R+++**	40	6	15.0
**Igbo-Abikou**	**R+++**	40	9	22.5
**Idena3**	**R+++**	40	11	27.5
**Alabansa**	**R+++**	40	7	17.5
**Tchaada**	**R+++**	40	2	05.0
**Adjozoume**	**R+++**	40	4	10.0
**Iwaï**	**R+++**	40	2	05.0
**Total (R+++)**		480	83	**17.3**
**Djohounkolé**	**R+**	40	6	15.0
**Kétougbékon**	**R+**	40	4	10.0
**Lokossa**	**R+**	40	13	32.5
**Itakpako**	**R+**	40	22	55.0
**Igbola**	**R+**	40	15	37.5
**Ita-soumba**	**R+**	40	23	57. 5
**Ko-Aïdjedo**	**R+**	40	9	22.5
**Zihan**	**R+**	40	18	45.0
**Gblo-Gblo**	**R+**	40	15	37.5
**Okéola**	**R+**	40	3	7.5
**Idena2**	**R+**	40	5	12.5
**Kpankoun**	**R+**	40	5	12.5
**Daagbe**	**R+**	40	3	7.5
**Total (R+)**		520	**141**	**27.1**

The mean haemoglobin rate in children was 9.4 g/dl (9.3 -9.5) without variation according to different localities (Table 2). Table 5 describes haemoglobin rates among children aged six to 59 months in areas of high and low resistance. In the 1,000 children assessed, 77% were anaemic. Eight on ten children that were aged six - 30 months against seven on ten of those aged 31–59 months had anaemia (Table 5). The anaemia observed in the six to 30 month old children was significantly higher than in the 31–59 month old children (p = 0.00) but no difference associated with resistance areas was observed (p = 0.35).

**Table 5:** Distribution of children’s haemoglobin rate between low and high resistance areas

	**Haemoglobin rate <11 g/dl**			
**Age (months)**	**Low resistance area**		**High resistance area**	
	**Number (%)**	**Total population**	**Number (%)**	**Total population**
**[6-30] months**	244 (80.3)	304	256 (86.5)	296
**[31–59] months**	146 (67.8)	216	143 (77.7)	184
**Total**	390 (75.0)	520	399 (83.1)	480

For age: p = 0.00 For area resistance: p = 0.35.

### Effect of resistance on LLIN effectiveness

The risk of having malaria is significantly higher for children who did not sleep under LLINs than for children who do in the two areas (Table 6). But the prevalence of malaria was higher among children that used LLINs in areas with low resistance than in areas with high resistance. A similar result was observed with children that did not use LLINs in the areas. The risk of having malaria was significantly reduced (p < 0.05) with LLIN use in both low and high resistance areas. The preventive effect of LLINs in high resistance areas was 60% (95% CI: 40–70), and was significantly higher than that observed in low resistance areas (p < 0.05).

**Table 6:** Effect of resistance on LLIN effectiveness by infection prevalence

						**LLIN use**	**Effect modification of resistance on effectiveness**
			**Total positive**	**Total tested by microscopy**	**Prevalence of *P. f *** **(%)**	**Odds ratio LLIN no use *versus *** **use net**	**Odds ratio, low *versus *** **high resistance**
**Low resistance**	LLINs	No	46	124	37.1 (36.3-38.0)	1	
		Yes	89	356	25.0 (24.6-25.5)	1.8 (1.1-2.8) p = 0.00	1
**High resistance**	LLINs	No	46	166	27.7 (27.0-28.4)	1	
		Yes	43	354	12.1 (11.8-12.4)	2.8 (1.7-4.5) p = 0.000	0.4 (0.3 0.6) p = 0.000

Table 7 shows that the use of LLINs reduces the prevalence of anaemia in both low and high insecticide resistance areas. Anaemia was significantly higher in children who did not use LLINs compared to children who used them, in areas of low resistance (p = 0.02), whereas in high resistance areas the risk was not significant (p = 0.67). The prevalence of anaemia associated with LLIN use was significantly higher in areas with low resistance than in areas with high resistance (p = 0.000).

**Table 7:** Effect of resistance on LLIN effectiveness by prevalence of anaemia

**Resistance area**	**LLIN use**	**Anaemia +**	**Anaemia -**	**Total**	**Anaemia Prevalence (%)**	**Odds ratio LLIN no use *versus *** **use net**	**Odds ratio high *versus *** **low resistance**
**Low resistance**							
	No	54	19	73	74.0	1	
	Yes	345	62	407	84.8	0.5 (0.3-0.9)	1
**Total low resistance**		**399**	**81**	**480**	**83.1**	p = 0.02	
**High resistance**	No	85	26	111	76.6	1	
	Yes	305	104	409	74.6	1.1	1.9 (1.3-2.7)
**Total high resistance**		**390**	**130**	**520**	75.0		
**Total**		**789**	**211**	**1,000**	**78.9**	p = 0.67	p = 0.000

## Discussion

The results of LLIN effectiveness in malaria prevention in vector resistance area showed that the resistance of vectors does not reduce the effectiveness of LLINs, but the prevalence of malaria and anaemia was higher in low resistance areas, and was in contradiction with what was expected. *Anopheles gambiae*, the main vector of malaria in Africa, has developed a strong resistance to pyrethroid in southern Benin [25]. This resistance has been observed not only in urban areas and in areas characterized by cotton growing but also in rural areas where traditional farming does not require the use of agricultural insecticides or fertilizers [11,15,26]. The main mechanism of pyrethroid resistance observed in southern Benin is based on the modification of target in the vectors. Contrary to that observed in some African countries, such as Burkina Faso [27], this resistance is high in *An. gambiae* M and S form. The M form was the predominant population in southern Benin in general, and particularly in this study area. These results confirm those of Yadouléton *et al. *[13] showing that the resistance of malaria vectors to insecticides was growing in Benin.

In order to determine the influence of pyrethroid resistance on LLIN efficacy, the evolution of vector susceptibility in the study area was monitored. Survey results showed that phenotypic resistance varied strongly over time when compared with 2011 data [26]. This variation has led to recommendations for the WHO village classification. Indeed, median value of the deltamethrin mortality was used for clustering of villages of high and low resistance. The median value for mortality in this study was 79%. This suggested that the mortality induced by deltamethrin has decreased. So, vector susceptibility to deltamethrin appears a dynamic phenomenon, which could be influenced either by intra- and extra-parameters, such as climatic conditions, ecological factors, or season.

*Kdr* mutation is responsible of pyrethroid resistance but detoxification mechanisms are also involved. Until now, the part of each mechanism does not know in the phenotypes observed in this study. *Kdr* results showed that there was a significant difference between the low and high resistance villages in 2012. The frequencies of this mutation are significantly lower in low resistance areas than in high resistance areas. The mutation was also found either in the M and S form. This could be explained by a high selection pressure of the *kdr* gene in the field populations of vectors. Therefore, the correlation between phenotypic resistance (susceptibility to deltamethrin) and genotypic resistance does not observe [27]. The metabolic mechanisms involved in pyrethroid resistance are present in Benin [28-31]; complementary studies on these genes should be conducted to address this question.

The LLIN coverage of households in children provenance in this study (88%) and the utilization rate of LLINs by children (71%) were better. Furthermore, no significant difference was observed between the coverage and the usage of LLIN in both localities ( R + and R + + + ). Thus, both arms have been homogeneity and these factors do not affect the analysis of results.

The prevalence of malaria parasitaemia in this study population was 22%, and variations were found between clusters (5.0-57.5). It was lower than the 44.4% prevalence reported in children < five years of age from the malaria indicator survey conducted in the same region in 2010 [32]. These prevalences were similar to those observed by Pond [33] among children living in rural communities distant by 150 km to cities or within the same zone of malaria endemicity. This study showed that in 14 of 20 large cities, all the children living in 75% or more of the clusters were malaria parasite-free. The decrease in the prevalence of malaria parasites may be due to the control measures recently implemented by the Benin Government through the Ministry of Public Health [34]. The measures include a nationwide free distribution of LLINs [10]. The decline in malaria burden attributed to the use of interventions such as LLINs was also reported in malaria-endemic countries, such as Kenya [35].

The prevalence of anaemia in this population of young infants was 78.9%, nearly identical to those rates reported (79%) for the region in the malaria indicator survey conducted in 2010 [31]. The prevalence of anaemia observed in the study is not unexpected as a positive relationship with resistance. The level of haemoglobin (<11 g/dl) used as an indicator of anaemia was not significantly influenced by vector resistance to insecticide. Achidi *et al.*[36] in Cameroun showed that the difference of prevalence of anaemia was not unexpected in the locality. They could potentially reflect the decline of nutritional status.

In this study, LLIN effectiveness in malaria prevention was significantly higher in the resistance area. The prevention of anaemia by the use of LLINs was also higher in areas of high resistance. According to a recent study on malaria transmission in the study area [37], vector density was very high in low resistance areas. These authors noted in low-resistance area a high EIR of 184.5 infected bites /man /6 months against 66.7 infected bites /man /6 months ( p <0.001) in the high resistance area. Similarly, the prevalence of malaria infection was 27.1% in low resistance area against 17.3% in high resistance area. However, no significant difference was observed between the prevalence of anemia in two areas. The high level of transmission obtained in the region should thus lead to a greater number of malaria cases. The results of a recent study [38] suggest that feeding on human hosts whose blood has been depleted due to severe anaemia did not significantly reduce the ability or potential transmission of malaria vectors, and indicates that mosquitoes may be able to exploit the few resources from a low level of haemoglobin rather than one that is normal in order to reproduce. For proper evaluation of the impact of vector resistance to pyrethroids on the effectiveness of LLINs, it would be desirable to have two frankly different areas of susceptibility vectors status: one where the Anopheles was resistant and another one where Anopheles was fully susceptible. In addition, the two areas must have the same ecological characteristics. Unfortunately, the sharp increase in the vectors resistance in southern Benin, has not allowed us to obtain such areas and this is what constitutes the main limitation of this study. Another limitation of this study was the cross-sectional study design. Associations presented could have been confounded by unmeasured factors and therefore causal inferences cannot be drawn. In addition, the temporal relationship between exposure variables (evolution of resistance vectors, the effectiveness of the use LLIN) and outcomes of interest (occurrence of malaria cases and other related factors) cannot be observed. Finally, because this study enrolled participants using convenience sampling and was done in a single geographically defined area, care should be taken in generalizing the results to the other populations.

## Conclusion

In the surveyed study area, resistance of malaria vectors seem to date not have affected the impact of LLINs and the use of LLINs was highly associated with reduced malaria prevalence irrespective of resistance. The surprising result of lower prevalence in high resistance areas is likely due to differences in mosquito populations, e.g. larval habitat distribution, productivity and adult density but that there should be further studies to determine the possible causes of such results.

## Competing interests

The authors declare that they have no competing interests.

## Authors’ contributions

FTT, MCA, AM, DKG, AHO, and IK participated in the coordination of the study, data analysis and manuscript preparation. AAA, AW and DG participated in coordination of the study, data collection and manuscript preparation. VG helped in mapping, manuscript preparation and revision. OM participated in the coordination of the study and data collection. YS, RO, AS and SC participated in the study design and manuscript preparation. All authors read and approved the final manuscript.
